# Characteristics of Inpatient Falls and Factors Associated with Fall-Related Fractures and Recurrent Falls in a Japanese University Hospital

**DOI:** 10.3390/medsci14030410

**Published:** 2026-07-21

**Authors:** Norio Imai, Yoji Horigome, Daisuke Homma, Hiroyuki Kawashima

**Affiliations:** 1 Division of Comprehensive Musculoskeletal Medicine, Graduate School of Medicine, Dentistry and Health Sciences, Niigata University, Niigata 951-8510, Japan; 2 Division of Orthopedic Surgery, Department of Regenerative and Transplant Medicine, Graduate School of Medical and Dental Sciences, Niigata University, Niigata 951-8510, Japan

**Keywords:** fall, fall-related fracture, recurrent fall, university hospital, associated factor

## Abstract

Background: Inpatient falls are among the most common adverse events in acute-care hospitals and may result in functional decline, prolonged hospitalization, and increased healthcare costs. However, the characteristics of fall-related fractures and recurrent falls in university hospitals have not been fully investigated. Methods: We retrospectively reviewed all inpatient fall events reported to the Department of Patient Safety at Niigata University Medical and Dental Hospital between January 2021 and December 2025. Patients younger than 20 years were excluded. Data regarding patient characteristics, mobility status, fracture occurrence, recurrent falls, medication use, and clinical departments were collected from medical records. Univariate and multivariable logistic regression analyses were performed to identify factors associated with fall-related fractures and recurrent falls. Results: A total of 2571 fall events were identified, yielding an incidence of 2.306 falls per 1000 inpatient-days. Malignant neoplasms were the most common primary diagnosis (36.9%). The incidence of falls was highest in the Departments of Neurology and Neurosurgery. Fall-related fractures occurred in 54 cases (2.1%), most commonly involving the lower extremities, including 17 proximal femoral fractures. Multivariable analysis identified mobility status as the only independent factor associated with fractures (odds ratio [OR], 1.821; 95% confidence interval [CI], 1.263–2.627; *p* = 0.001). Receiver operating characteristic analysis demonstrated a mobility cutoff value of 2.5, indicating an increased fracture risk among patients who were ambulatory with assistive devices or had higher mobility levels. Recurrent falls were observed in 439 patients (24.2%). Reduced mobility (OR, 0.837; 95% CI, 0.713–0.981; *p* = 0.028), polypharmacy (≥5 medications) (OR, 1.634; 95% CI, 1.254–2.016; *p* < 0.001), and use of central nervous system depressants (OR, 1.363; 95% CI, 1.124–1.653; *p* = 0.002) were independently associated with recurrent falls. Conclusions: Patients with fall-related fractures tended to have relatively high mobility, whereas recurrent falls were associated with reduced mobility, polypharmacy, and central nervous system depressant use. Different characteristics of falls were observed across clinical departments. These findings may help identify patients at high risk for adverse fall-related outcomes and support the development of targeted fall-prevention strategies in university hospitals.

## 1. Introduction

Inpatient falls are among the most common adverse events in acute care hospitals and are associated with a decline in activities of daily living (ADL), prolonged hospitalization, and increased healthcare costs [1,2,3,4,5]. Morello et al. reported that patients who experienced inpatient falls had an average hospital stay that was 8 days longer and incurred an additional healthcare cost of AUD 6669 compared with patients who did not fall. Furthermore, among patients who sustained fall-related injuries, the length of hospital stay increased by an average of 11 days and the additional healthcare cost reached AUD 9917 [4]. In particular, falls resulting in fractures have a substantial impact on patient outcomes, including increased mortality, and their clinical importance is expected to increase further with population aging [5,6,7]. Despite these studies, relatively little is known about the characteristics of fall-related fractures and recurrent falls in tertiary university hospitals.

Although numerous studies have investigated the epidemiology, risk factors, and prevention strategies of inpatient falls [1,8,9], few have comprehensively examined the characteristics of fall-related fractures and recurrent falls according to clinical departments in a university hospital setting. University hospitals often provide specialized care for patients with neurological disorders, malignancies, and hematologic diseases, resulting in patient populations that differ substantially from those in general acute-care hospitals [9]. Therefore, the characteristics and risk factors of inpatient falls in university hospitals may differ from those observed in general acute care hospitals.

The aim of this study was to investigate the characteristics of patients who experienced inpatient falls and to identify factors associated with fall-related fractures and recurrent falls among these patients. A better understanding of the clinical profiles of patients at high risk for fractures and recurrent falls may facilitate the implementation of targeted preventive interventions and improve patient safety in the hospital setting.

## 2. Materials and Methods

### 2.1. Participants

This retrospective study included all inpatient fall events reported to the Department of Patient Safety at Niigata University Medical and Dental Hospital between January 2021 and December 2025. Patients younger than 20 years were excluded because the epidemiology and contributing factors of falls may differ between pediatric and adult populations.

Niigata University Medical and Dental Hospital is a tertiary referral university hospital with approximately 780 beds and functions as an advanced acute-care center providing tertiary emergency medical services.

### 2.2. Survey Items

The incidence of inpatient falls was calculated by dividing the total number of fall events by the cumulative inpatient-days (defined as the sum of the daily inpatient census during the study period) and expressed as the number of falls per 1000 inpatient-days (‰).

Data on age, sex, primary diagnosis at admission, mobility status at the time of the fall, location of the fall, activity at the time of the fall, causes of the fall, presence and site of fractures, dementia status, number of prescribed medications, use of central nervous system (CNS) depressants, and use of benzodiazepines were collected from the medical records.

The number of medications was analyzed not only as a continuous variable, in addition, based on the report by Kojima et al. [10] involving Japanese older adults, patients were categorized into two groups according to the presence of polypharmacy (<5 medications vs. ≥5 medications).

Fall events were categorized into three time periods based on the standard nursing shift schedule in Japan: early morning (00:00–07:59), daytime (08:00–16:59), and nighttime (17:00–23:59).

Primary diagnoses at admission were categorized into eight groups: cardiovascular, respiratory, renal/metabolic, gastrointestinal, neurological/psychiatric, musculoskeletal, malignant neoplasms, and others.

Departments were categorized as Cardiovascular Medicine, Endocrinology and Metabolism, Hematology, Nephrology and Rheumatology, Respiratory Medicine, Gastroenterology, Neurology, Medical Oncology, Psychiatry, Gastrointestinal Surgery, Breast and Endocrine Surgery, Cardiovascular Surgery, Thoracic Surgery, Orthopaedic Surgery, Plastic Surgery, Neurosurgery, Dermatology, Urology, Ophthalmology, Otolaryngology, Obstetrics and Gynecology, and Emergency Medicine.

Mobility status was classified into five categories: bedridden, requiring minimal assistance for transfer or ambulation, ambulatory with assistive devices, independent outdoor ambulation, and no mobility impairment. Each patient was assigned to the category that most closely reflected his or her mobility status at the time of the fall. Mobility status was retrospectively determined by reviewing the electronic medical records and nursing records at the time of the fall. The classification was performed by a single investigator according to the predefined five-category classification described above, based on the documented functional status at the time of the fall. The causes of falls were classified into four categories: physical factors, medical factors (e.g., fever, dizziness, and arrhythmia), cognitive/psychiatric factors, and environmental factors (e.g., tripping over objects or chair-related incidents). Because multiple factors may contribute to a single fall event, more than one cause could be assigned to each case.

The proportion of recurrent fallers was calculated using the number of patients who experienced two or more falls as the numerator and the total number of patients who experienced at least one fall as the denominator. Patients with recurrent falls were counted only once regardless of the number of fall events.

### 2.3. Statistical Analysis

Statistical analyses were performed using IBM SPSS Statistics for Windows, version 28.0 (IBM Corp., Armonk, NY, USA). Continuous variables, including age, were compared using Student’s t-test, whereas categorical variables, including sex and the presence of dementia, were analyzed using the chi-square test. Fisher’s exact test was used when the expected cell count was less than five.

Univariate analyses were performed to identify factors associated with fall-related fractures and recurrent falls. Variables showing statistical significance in the univariate analyses were subsequently entered into multivariable logistic regression models to identify independent associated factors.

Receiver operating characteristic (ROC) curve analysis was performed to determine the optimal cutoff value of mobility status for predicting fall-related fractures and recurrent falls.

A two-sided *p* value of <0.05 was considered statistically significant.

### 2.4. Ethics Statement

This study was approved by the Ethics Committee of Niigata University (Approval No. 2025-0336). Owing to the retrospective and non-interventional nature of the study, informed consent was waived and an opt-out procedure was used.

## 3. Results

**Figure 1:**
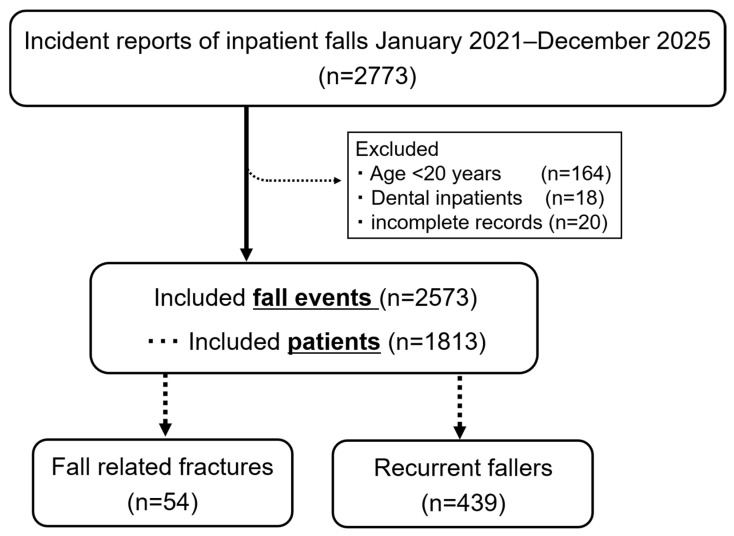
Flowchart of patient selection and study population. Dotted arrows indicate separate analytical pathways, and the ellipsis (…) indicates that the included fall events were used for both analyses.

A total of 2571 inpatient fall events were identified during the study period (Figure 1), yielding an incidence rate of 2.306 falls per 1000 inpatient-days. The mean patient age was 69.0 ± 13.2 years (Table 1).

Malignant neoplasms were the most common primary diagnosis at admission (949 cases), followed by neurological/psychiatric disorders (636 cases) and cardiovascular diseases (246 cases) (Table 1). Falls most frequently occurred in patient rooms (883 cases) and toilets (269 cases), and toileting was the most common activity at the time of the fall (976 cases) (Table 1). Physical impairment was the most frequently identified contributing factor (1126 cases) (Table 1).

**Figure 2:**
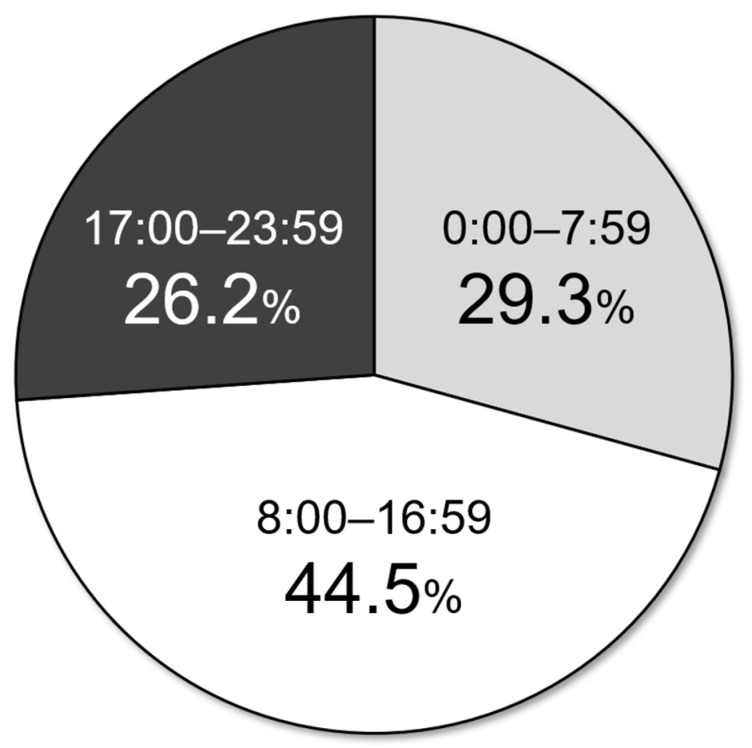
Distribution of inpatient falls according to time of occurrence. The daytime period accounted for the highest proportion of events, whereas the nighttime period accounted for the lowest proportion.

Regarding the timing of falls, the daytime period accounted for the highest proportion of events (44.5%), whereas the nighttime period accounted for the lowest proportion (26.2%) (Figure 2).

**Figure 3:**
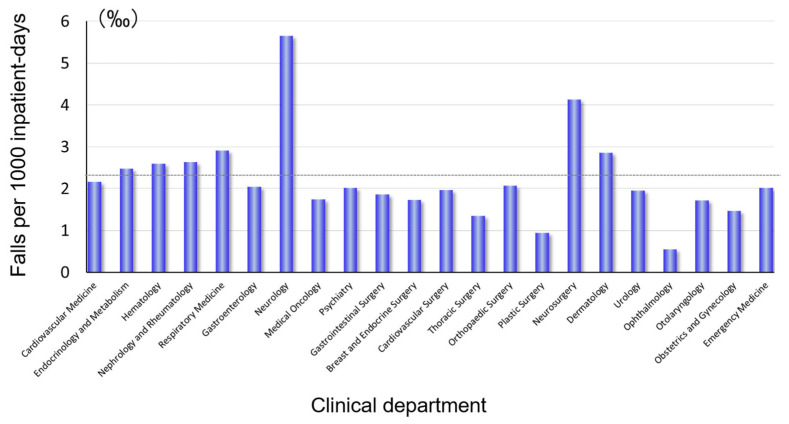
Incidence of inpatient falls by clinical department. The incidence of falls was highest in the Departments of Neurology and Neurosurgery, followed by the Departments of Respiratory Medicine and Dermatology. The dashed line indicates the overall hospital average.

The incidence of falls was highest in the Departments of Neurology and Neurosurgery, followed by the Departments of Respiratory Medicine and Dermatology (Figure 3).

**Figure 4:**
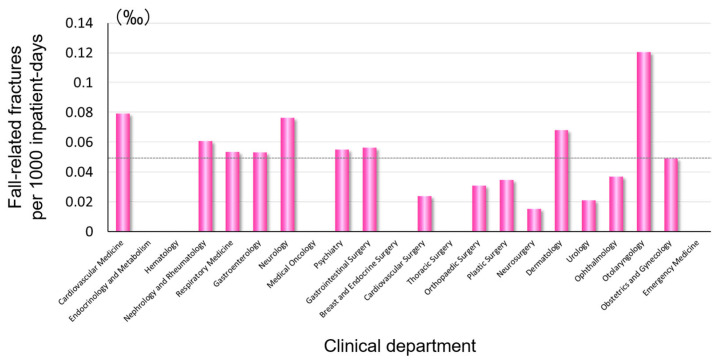
Incidence of fall-related fractures by clinical department. The incidence of fall-related fractures was highest in the Department of Otolaryngology, followed by the Departments of Cardiovascular Medicine and Neurology. The dashed line indicates the overall hospital average.

Fall-related fractures occurred in 54 cases (2.1%) (Table 2). Lower-extremity fractures were the most common type (22 cases), including 17 proximal femoral fractures (Table 2). The incidence of fall-related fractures was highest in the Department of Otolaryngology, followed by the Departments of Cardiovascular Medicine and Neurology (Figure 4).

**Figure 5:**
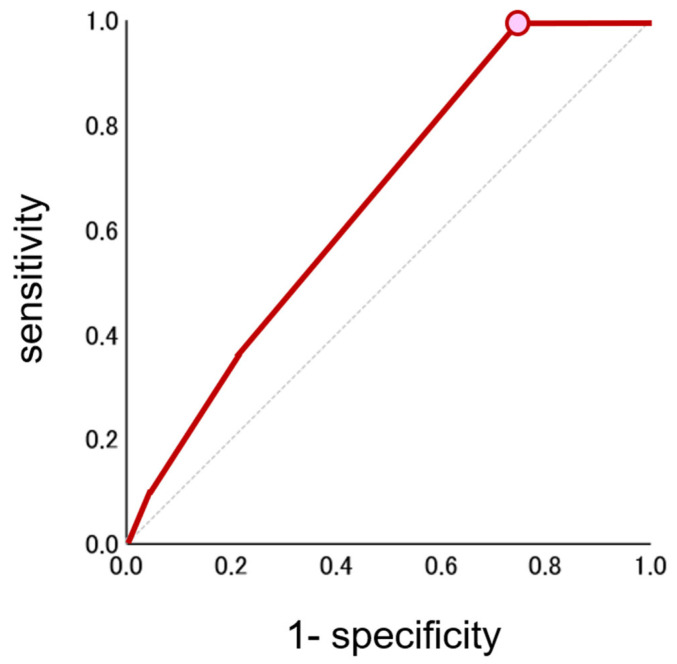
Receiver operating characteristic curve (ROC) analysis for fall-related fracture. The circle indicates the optimal cutoff value of mobility status (2.5), and the dashed diagonal line represents the line of no discrimination (AUC = 0.5).

In the univariate analysis, female sex, mobility status, number of medications, and use of CNS depressants were significantly associated with fall-related fractures (Table 3). In the multivariable logistic regression analysis, Mobility status was the only independent factor identified in the present analysis (odds ratio [OR], 1.821; 95% confidence interval [CI], 1.263–2.627; *p* = 0.001); however, this association should be interpreted with caution because important determinants of bone fragility were not available for adjustment. ROC analysis identified an optimal cutoff value of 2.5 for mobility status, indicating that patients who were ambulatory with assistive devices or had higher mobility levels were at increased risk of fractures (Figure 5).

**Figure 6:**
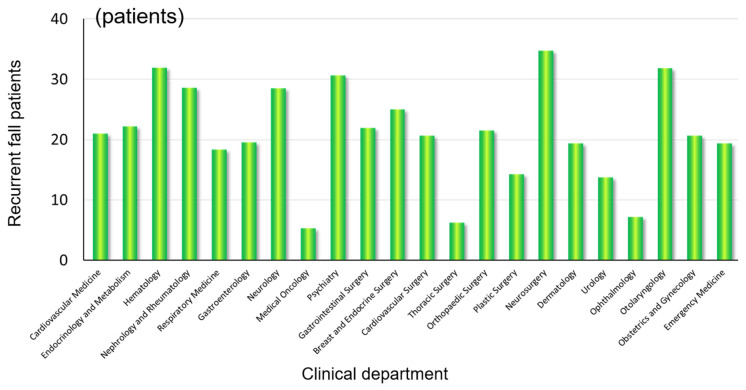
Incidence of recurrent falls by clinical department. The incidence of recurrent falls was highest in the Department of Neurosurgery, followed by the Departments of Otolaryngology, Hematology, and Psychiatry.

Among the 439 patients with recurrent falls, 3 (0.7%) sustained fractures, including one proximal humeral fracture, one distal radius fracture, and one proximal femoral fracture (Table 4). Fisher’s exact test demonstrated that recurrent fallers were significantly less likely to sustain fractures than patients who experienced a single fall (0.7% vs. 3.7%, *p* = 0.001). The highest incidence of recurrent falls was observed in the Department of Neurosurgery, followed by the Departments of Otolaryngology, Hematology, and Psychiatry (Figure 6).

In the univariate analysis, falls occurring during activities of daily living, reduced mobility, increased number of medications, and use of CNS depressants were significantly associated with recurrent falls (Table 5). Multivariable logistic regression analysis identified reduced mobility (OR, 0.837; 95% CI, 0.713–0.981; *p* = 0.028), polypharmacy (≥5 medications) (OR, 1.634; 95% CI, 1.254–2.016; *p* < 0.001), and use of CNS depressants (OR, 1.363; 95% CI, 1.124–1.653; *p* = 0.002) as independent factors associated with recurrent falls.

**Figure 7:**
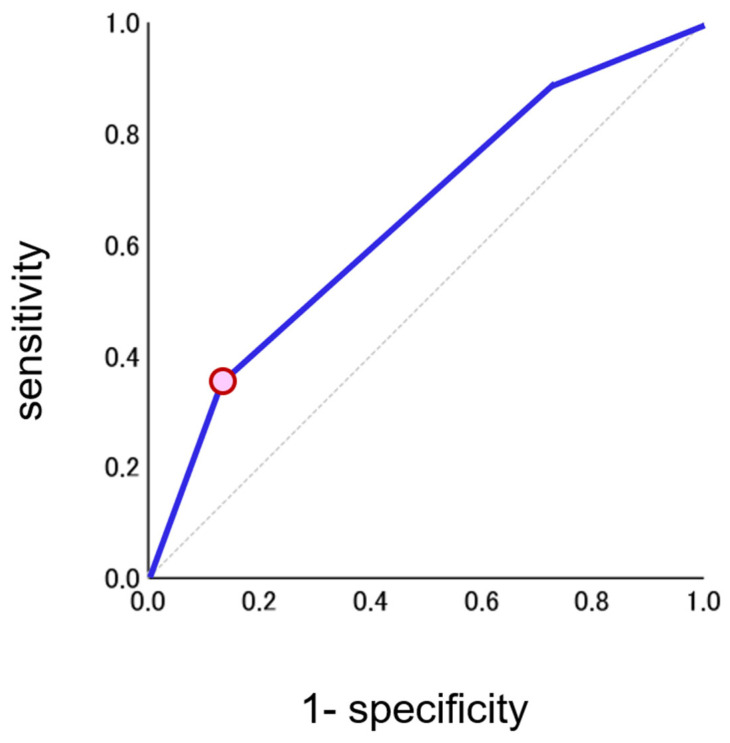
Receiver operating characteristic (ROC) curve analysis for recurrent falls. The circle indicates the optimal cutoff value of mobility status (3.5), and the dashed diagonal line represents the line of no discrimination (AUC = 0.5).

ROC analysis identified an optimal cutoff value of 3.5 for mobility status, indicating that patients with mobility levels of ambulatory with assistive devices or lower were at increased risk of recurrent falls (AUC, 0.553; 95% CI, 0.522–0.583; *p* = 0.001) (Figure 7).

## 4. Discussion

In the present study, we analyzed 2571 inpatient fall events that occurred at Niigata University Medical and Dental Hospital. The overall incidence of falls was 2.306 per 1000 inpatient-days, which was comparable to previously reported rates of 2 to 7 falls per 1000 inpatient-days in acute care hospitals, including the average rate reported in Japan (2.83 per 1000 inpatient-days) [1,8,11]. However, several findings appeared to reflect the unique patient population of a university hospital, including department-specific characteristics [12] as well as distinctive features of patients who sustained fall-related fractures or experienced recurrent falls.

The incidence of falls was highest in the Departments of Neurology and Neurosurgery, consistent with previous reports [8,9,11]. Patients with neurological disorders often possess multiple risk factors for falls, including motor weakness, gait disturbance, balance impairment, cognitive dysfunction, and attentional deficits. In addition, the frequent use of antiepileptic and psychotropic medications may further increase fall risk [8,9]. Fischer et al. reported that patients with neurological disorders have high rates of both initial and recurrent falls [9], and our findings are in agreement with these observations.

The relatively high incidence of falls observed in the Departments of Respiratory Medicine and Dermatology was also noteworthy. A possible explanation is that patients with respiratory diseases are often older and may experience impaired physical function due to dyspnea, hypoxemia, sarcopenia, and frailty associated with chronic respiratory conditions such as chronic obstructive pulmonary disease (COPD) and interstitial lung disease [13,14]. Furthermore, oxygen tubing and intravenous lines may serve as environmental risk factors for falls [15]. Previous studies have reported a higher incidence of falls among patients with COPD than among community-dwelling older adults, and our findings may reflect these underlying characteristics of respiratory patients [13].

One possible explanation is that the high incidence of falls among dermatology patients may be attributable to the unique patient population encountered at a university hospital. At our institution, many dermatology inpatients are older adults with severe conditions such as bullous pemphigoid, pemphigus, severe drug eruptions, and chronic leg ulcers. These patients often experience activity restrictions due to pain and wound protection, in addition to muscle weakness associated with high-dose corticosteroid therapy and immunosuppressive treatment [16,17]. Moreover, activity limitations related to extensive skin lesions, wound dressings, and chronic ulcers, as well as the use of hypnotics or psychotropic medications prescribed for pruritus-related sleep disturbances, may contribute to an increased risk of falls [18]. Patients with bullous pemphigoid are also frequently older and have a higher prevalence of dementia and other neurological disorders, which may further increase fall risk [18,19].

With regard to the circumstances of falls, toileting was the most common activity at the time of the event, and most falls occurred in patient rooms or toilets. These findings are consistent with previous reports from both Japan and other countries [8,11,15]. Factors such as urinary urgency leading to hurried ambulation, unassisted bed exits, reduced visibility during nighttime hours, orthostatic hypotension, and the effects of hypnotic medications may have contributed to these falls [8,9,15]. Furthermore, because physical impairment was the most frequently identified contributing factor, our findings underscore the importance of appropriate toileting assistance and mobility support for fall prevention.

One of the notable findings of this study was that patients with malignant neoplasms constituted the largest disease category among those who experienced falls. In previous studies of inpatient falls, neurological disorders and elderly medical patients have typically represented the predominant patient populations [8]. However, university hospitals provide advanced oncological care and therefore manage a large number of patients with malignancies [9]. Recent studies have demonstrated that sarcopenia, frailty, cancer cachexia, and chemotherapy-induced peripheral neuropathy are important risk factors for falls among patients with cancer [20,21,22]. In particular, the concept of “cancer locomotive syndrome (cancer loco-motive syndrome),” proposed by the Japanese Orthopaedic Association, has drawn attention to the decline in physical function and increased risk of falls associated with cancer and its treatment [23,24,25]. Furthermore, the use of opioids, hypnotics, and other psychotropic medications may further increase fall risk [20,21,26]. These findings suggest that effective fall-prevention strategies in university hospitals should include careful assessment and targeted interventions for patients with malignant neoplasms.

A total of 54 patients sustained fall-related fractures, with lower-extremity fractures, particularly proximal femoral fractures, being the most common. In the multivariable analysis, mobility status was identified as the only independent factor associated with fall-related fractures. In general, risk factors for falls and those for fall-related fractures do not necessarily overlap. While falls themselves are more common among patients with impaired ADL, severe injuries and fractures tend to occur in patients who retain a certain level of mobility, as ambulatory patients are more likely to fall from a standing position and sustain higher-energy injuries [27].

In the present study, patients with fractures tended to have higher mobility levels and lower rates of central nervous system depressant use and fewer prescribed medications than those without fractures. These findings suggest that patients who are prone to falling are not necessarily the same as those who are prone to sustaining fractures. Patients with limited mobility often experience low-energy falls occurring at the bedside or during short-distance transfers, whereas ambulatory patients are more likely to fall while standing or walking, resulting in greater impact energy and an increased likelihood of fracture. Our findings are therefore consistent with previous reports [27].

Furthermore, ROC analysis identified “ambulatory with assistive devices” as the threshold associated with fracture occurrence. However, the discriminatory performance was modest (AUC = 0.611), indicating that mobility status alone has limited clinical utility for predicting fall-related fractures.

The incidence of fall-related fractures was highest in the Department of Otolaryngology. The relatively high fracture incidence observed in the Department of Otolaryngology may partly reflect the characteristics of the patient population treated at our institution, including patients with head and neck cancer, who often experience malnutrition, sarcopenia, and functional decline associated with chemoradiotherapy [21,22,23,24,25]. However, because these disease-specific characteristics were not directly evaluated in the present study, this explanation should be considered hypothesis-generating. In addition, this patient population includes individuals with vestibular dysfunction and dizziness. Although many of these patients retain a relatively high level of mobility, concomitant balance impairment may predispose them to sudden falls and increase the likelihood of sustaining fractures [10,28,29]. Previous studies have also reported an increased risk of proximal femoral fractures among patients with vestibular disorders and balance impairment [10].

Recurrent falls accounted for 24.2% of all fall events. Multivariable analysis identified reduced mobility, polypharmacy (≥5 medications), and the use of CNS depressants as independent factors associated with recurrent falls. Similarly, although ROC analysis identified a mobility cutoff associated with recurrent falls, the discriminatory ability was poor (AUC = 0.553). Therefore, this cutoff should be interpreted with caution and should not be used as a stand-alone tool for clinical prediction. Polypharmacy is a major challenge in geriatric medicine and has been widely recognized as a risk factor for falls [30,31,32]. In particular, CNS depressants and benzodiazepines may increase the risk of recurrent falls by causing sedation, impaired attention, orthostatic hypotension, and gait disturbances [1,33]. Our findings further emphasize the importance of regular medication review and optimization as part of inpatient fall-prevention strategies. Among the 439 recurrent fallers, only three patients (0.7%) sustained fractures, a significantly lower proportion than that observed among single-fall patients. This finding suggests that patients with recurrent falls and those who sustain fall-related fractures may have different clinical characteristics. However, because only three recurrent fallers sustained fractures, this observation should be interpreted with caution and confirmation in larger studies is required. Recurrent falls tend to occur in patients with impaired mobility and low-energy falls, whereas fractures may be more likely to occur in relatively active patients who sustain higher-energy falls while standing or walking.

Department-specific analysis revealed that recurrent falls were particularly common among patients in the Departments of Neurosurgery, Psychiatry, and Hematology. Neurosurgical patients often have motor weakness, higher cortical dysfunction, attentional deficits, and impaired awareness of their own limitations. Furthermore, neurological impairment has been identified as one of the strongest predictors of recurrent falls [9,34,35].

Psychiatric patients frequently have schizophrenia, bipolar disorder, dementia, or delirium, all of which may impair judgment, attention, and mobility. In addition, the use of antipsychotics, benzodiazepines, and hypnotics may further increase the risk of recurrent falls [1,36,37].

Patients in hematology departments often have anemia, chemotherapy-related adverse effects, prolonged bed rest, frailty, and sarcopenia. Recent studies have demonstrated that frailty and impaired physical function are highly prevalent among patients with hematologic malignancies and are associated with an increased risk of falls. Hematologic malignancies themselves have also been reported to be closely associated with frailty and falls [38,39]. The proposed explanations for department-specific differences should be regarded as hypothesis-generating rather than definitive.

## 5. Limitations

This study has several limitations. First, this was a single-center retrospective study conducted at a university hospital in Japan, which may limit the generalizability of our findings. Second, because the analysis was based on an incident reporting system, minor falls may have been underreported. Third, several clinically important variables, including bone mineral density, osteoporosis diagnosis, previous fragility fractures, vitamin D status, sarcopenia, muscle strength, comorbidity burden, nutritional status, frailty, visual impairment, laboratory findings, anti-osteoporotic treatment, and detailed medication profiles, were unavailable for analysis. Therefore, residual confounding cannot be excluded, and the observed association between mobility status and fall-related fractures should be interpreted with caution. Fourth, only patients who experienced inpatient falls were included, and no comparison was made with patients who did not fall. Therefore, the identified factors should not be interpreted as predictors of inpatient falls themselves but rather as factors associated with fall-related fractures and recurrent falls among patients who had already experienced inpatient falls. In addition, mobility status was retrospectively determined by reviewing electronic medical records and nursing records, and interobserver reliability of this classification was not formally evaluated. Finally, only three recurrent fallers sustained fractures, which limited our ability to perform a detailed analysis of the relationship between recurrent falls and fracture occurrence. Furthermore, comparisons among clinical departments were not adjusted for differences in patient volume, disease severity, length of hospital stay, or baseline patient characteristics. Therefore, the observed department-specific differences should be interpreted as descriptive findings reflecting differences in case mix rather than direct comparisons of departmental fall risk.

## 6. Conclusions

Despite these limitations, this study provides novel insights into the characteristics of fall-related fractures and recurrent falls among patients who experienced inpatient falls in a Japanese university hospital. This study clarified the characteristics of inpatient falls in a university hospital setting. Patients who sustained fall-related fractures tended to have relatively high levels of mobility, whereas recurrent falls were associated with reduced mobility, polypharmacy, and the use of central nervous system depressants. In addition, distinct patterns of falls were observed among different clinical departments. These findings may contribute to the identification of patients at high risk of adverse fall-related outcomes and for developing targeted fall-prevention strategies in university hospitals. Future multicenter studies are needed to validate our findings and to investigate additional patient-related factors, including bone mineral density, muscle strength, frailty, and detailed medication profiles. Prospective studies are also warranted to determine whether department-specific fall-prevention strategies can reduce the incidence of fall-related fractures and recurrent falls in university hospitals.

## Figures and Tables

**Table 1:** Characteristics of inpatient falls (n = 2571).

Number of Fall Events	2571	
Fall Incidence (per 1000 inpatient-days)	2.306	
Age (years)	65.3 ± 14.4	
Sex (Male/Female)	1425/1146	
Primary Diagnosis at Admission, n (%)	Cardiovascular Diseases	246 (9.6)
	Respiratory Diseases	93 (3.6)
	Renal and Metabolic Diseases	160 (6.2)
	Gastrointestinal Diseases	105 (4.1)
	Neurological and Psychiatric Disorders	636 (24.7)
	Musculoskeletal Disorders	241 (9.4)
	Malignant Neoplasms	949 (36.9)
	Others	141 (5.5)
Location of Fall, n (%)	Patient Room	1887 (73.4)
	Toilet	269 (10.5)
	Bathroom	115 (4.5)
	Corridor	232 (9.0)
	Others	68 (2.6)
Activity at the Time of Fall, n (%)	Toileting	980 (38.1)
	Transfer/Ambulation	594 (23.1)
	Activities of Daily Living	729 (28.4)
	Accident	5 (0.2)
	Unknown	261 (10.2)
Contributing Factors *, n	Physical Factors	1852
	Medical Factors	461
	Cognitive/Psychiatric Factors	845
	Environmental Factors	203
	Unknown	3
Number of Medications	7.2 ± 3.6	
Polypharmacy (≥5 medications), n (%)	1977 (76.9)	
Use of CNS Depressants, n (%)	Yes: 1166 (45.4)	No: 1401 (54.6)
Use of Benzodiazepines, n (%)	Yes: 453 (17.6)	No: 2118 (82.4)

* Multiple contributing factors could be assigned to a single fall event. Abbreviations: CNS, central nervous system.

**Table 2:** Characteristics of patients with fall-related fractures (n = 54).

Number of Fracture Cases	54	
Percentage of fall events (%)	2.1	
Incidence among all hospitalized patients (per 1000 inpatient-days)	0.048	
Age (years)	71.1 ± 10.4	
Sex (Male/Female)	22/32	
Fracture site, n (%)	Rib	6 (11.1)
	Vertebra	6 (11.1)
	Pelvis	3 (5.6)
	Upper extremity	12 (22.2)
	- Humerus	5
	- Radius	5
	- Figer	2
	Lower extremity	22 (41.2)
	- Proximal femur17	17
	- Tibia	1
	- Patella	2
	- ankle	1
	Head/face	5 (9.3)

**Table 3:** Univariate analysis of factors associated with fall-related fractures.

Factors Associated with Fall-Related Fractures (Univariate Analysis)	Correlation Coefficient	*p* Value
Female sex	0.243	0.028
Mobility status	0.262	0.002
Number of medications	−0.241	0.037
Use of CNS depressants	−0.247	0.018

Abbreviation: CNS, central nervous system.

**Table 4:** Characteristics of recurrent fallers (n = 439).

Recurrent Fallers, n	439
Single-fall patients, n	1374
Percentage of all recurrent fallers (%)	24.2
Age (years)	69.2 ± 13.0
Sex (Male/Female)	254/185
Fracture cases, n (%)	3 (0.7%) - Humerus 1 - Radius 1 - Proximal femur 1

**Table 5:** Univariate analysis of factors associated with recurrent falls.

Factors Associated with Recurrent Falls (Univariate Analysis)	Correlation Coefficient	*p* Value
Mobility status	−0.262	<0.001
Number of medications	0.214	0.012
Polypharmacy (≥5 medications)	0.385	<0.001
Use of central nervous system depressants	0.314	<0.001

## Data Availability

The data presented in this study are available on reasonable request from the corresponding author. The data are not publicly available due to ethical restrictions and the protection of participant privacy.
